# Oral Iron for Prevention and Treatment of Rickets and Osteomalacia in Autosomal Dominant Hypophosphatemia

**DOI:** 10.1002/jbmr.3941

**Published:** 2019-12-31

**Authors:** Wolfgang Högler, Klaus Kapelari

**Affiliations:** ^1^ Department of Paediatrics and Adolescent Medicine Johannes Kepler University Linz Linz Austria; ^2^ Institute of Metabolism and Systems Research University of Birmingham Birmingham UK; ^3^ Department of Paediatrics and Adolescent Medicine University Hospital Innsbruck Innsbruck Austria

**Keywords:** OSTEOMALACIA AND RICKETS, MATRIX MINERALIZATION, PTH/VIT D/FGF23, DISORDERS OF CALCIUM/PHOSPHATE, IRON DEFICIENCY, CELL/TISSUE SIGNALING, ENDOCRINE PATHWAYS, DISEASES AND DISORDERS OF/RELATED TO BONE, BONE MATRIX

## Abstract

Autosomal dominant hypophosphatemia (ADH) causes rickets, osteomalacia, and taurodontism due to heterozygous mutations in FGF23, which inhibit the inactivation (cleavage) of the encoded protein, the hormone fibroblast growth factor 23 (FGF23). Iron deficiency increases FGF23 mRNA expression and recent evidence suggests that the recurrent, late‐onset, or waxing‐waning hypophosphatemic phenotype may be linked to synchronous variations in iron status. The fact that most adult symptomatic ADH patients are females during reproductive age supports the notion of a gene‐environmental interaction. Practically all symptomatic hypophosphatemic patients described in the recent literature were also iron deficient (with/without anemia) at presentation, when measured. Given its interaction with FGF23, correcting iron deficiency should therefore also correct FGF23 excess. Following the original report of successful phenotype reversal in an iron‐deficient ADH child using oral iron supplementation in 2015, more evidence has emerged that supports the use of the element iron to restore homoeostasis of the element phosphorus (in addition to its own). We put into perspective the recent evidence and add 14 years observational data on the original case that demonstrates the correlation of serum phosphorus and renal tubular phosphate reabsorption in mass per unit volume of glomerular filtrate (TmP/GFR) with serum ferritin. Presentation and relapse of ADH, 12 years apart, occurred during iron deficiency, and the onset of menstrual periods was associated with relapse. Here we propose management guidance for patients affected by ADH throughout the lifespan based on iron stores. Because ferritin correlates best with hypophosphatemia historically, and in long‐term observation of the originally treated case, it should be used as the monitoring tool and kept in the normal range. Women with ADH who are of reproductive age and other risk groups require supplementation with oral iron using WHO guidelines. Treatment of this form of FGF23 excess may not require phosphate and active vitamin D, or burosumab. © 2020 American Society for Bone and Mineral Research

## Introduction

Hypophosphatemia has many heritable or acquired causes.^1^ Pediatric and adult bone specialists with experience in rare diseases are accustomed to managing patients with X‐linked hypophosphatemia (XLH). However, autosomal dominant hypophosphatemia (ADH) is much less common; even large metabolic bone centers often have little to no clinical experience in managing this disorder. Despite being caused by defects in two different genes (*PHEX* versus *FGF23*, respectively), XLH and ADH have in common the excessive serum concentrations of the hormone fibroblast growth factor 23 (FGF23). FGF23 excess results in renal phosphate loss with subsequent hypophosphatemia, muscle weakness, and impaired mineralization. Such hypomineralization affects (i) hypertrophic chondrocytes in the growth plates, leading to rickets; (ii) newly formed osteoid during modeling and remodeling, leading to osteomalacia; and (iii) teeth, leading to taurodontism with thin enamel layer and dentinal defects.

Historical terminology is still being used in the bone field. Genetic disorders should not be named after their childhood manifestation (rickets) but carry an ageless name. Therefore, similar to XLH (which previously included “Rickets”, XLHR), we chose to use ADH (not ADHR) for a condition that affects more than just growth plates.

Despite similar phenotype and complications from FGF23 excess, treatment studies have only been conducted in XLH and not ADH. In particular the FGF23 antibody burosumab^2^ is licensed for use in XLH, but not for use in ADH, or any other rare condition with FGF23 excess. However, recent advances in the understanding of ADH pathophysiology suggest a new therapeutic, and in fact also preventative, option for the renal loss of the element phosphorus (P, atomic number 15) in individuals with ADH: the element iron (Fe, atomic number 26).

## Unraveling the Mechanism of Disease: the Interplay of Elements

In 1958, Winters and colleagues^3^ documented dominant inheritance of hypophosphatemic rickets, apparently the first description of ADH. It took until 1997 to formally recognize ADH as an entity, and two patterns of onset were described, one presenting in childhood and one with onset in adulthood.^4^

In the year 2000, mutations in the *FGF23* gene were discovered as the cause of ADH.^5^ These *FGF23* mutations (R176Q/W and R179Q/W) replace Arg residues within a subtilisin‐like proprotein convertase cleavage site (RXXR motif), leading to protease resistance of the intact FGF23 hormone and therefore prolonging its half‐life.^6^ The cleavage site and its association with the remarkable waxing and waning ADH phenotype has since become the focus of interest. The unique “waxing/waning,” “late‐onset,” or “recurring” ADH manifestations soon lead to speculation of a gene–environment association. In particular, late onset disease in women with ADH suggested an association with iron deficiency from menstrual blood loss or hormonal interference.^4^, ^7^

In 2011, Imel and colleagues^8^ reported an apparent correlation of the serum levels of iron and phosphate in symptomatic ADH patients, suggesting that iron deficiency increases intact FGF23, a feature not seen in controls. Hence, iron deficiency, by a yet unknown gene–environment interaction mechanism increases *FGF23* expression, probably on a transcriptional and posttranslational level. In the same year, these human results were replicated in an ADH mouse model. ADH mice had elevated intact Fgf23 levels and hypophosphatemia during iron deficiency, whereas wild‐type controls maintained normal serum intact Fgf23 and inorganic phosphate levels. Also, bone Fgf23 mRNA and serum c‐terminal Fgf23 were induced in all mice during iron deficiency.^9^ Further evidence linking iron deficiency and FGF23 overexpression comes from oral iron supplementation of anemic Gambian children where ferritin concentrations negatively correlated with plasma FGF23.^10^

In 2019, Liu and colleagues^11^ published a report on six Chinese ADH kindreds including 20 patients affected by R176Q/W and R179Q/W mutations. They showed that at least seven of the 11 symptomatic, hypophosphatemic patients were also iron deficient, and 90% were female. They also summarized all the medical literature, which now includes only 13 documented ADH kindreds, and found that patients with R179Q/W mutations appeared to present earlier in life than those with R176Q/W mutations, a finding that will have to be confirmed over time.

## Oral Iron Therapy Reverses the ADH Phenotype

In 2015, Kapelari and colleagues^12^ were the first to report an iron‐deficient girl with ADH and active rickets whose phosphate metabolism normalized and rickets fully healed on oral iron treatment alongside normalization in ferritin levels, allowing for rickets medication to be discontinued. In 2019, the Chinese group^11^ also reported improvement and normalization in phosphate metabolism in several symptomatic, iron‐deficient ADH patients using oral iron therapy but did not provide comprehensive treatment data.

In this issue of the *Journal of Bone Mineral Research* (*JBMR*), Imel and colleagues^13^ corroborate the role of oral iron in ADH reversal in a well‐conducted prospective study in five adults. Oral iron doses were titrated to serum iron concentrations and reached 130 to 260 mg/day at the end of the study. The study is remarkable because, despite its small sample size (*n* = 5), it depicts two distinctly different groups of patients. At baseline, the three hypophosphatemic subjects with the highest FGF23 concentrations were iron deficient and had a striking increase in serum phosphorus concentrations in response to oral iron therapy and only a mild increase in ferritin. This remarkable response was much in contrast to the two subjects who at baseline had normal FGF23, phosphorus, and ferritin levels and showed no clinically meaningful response in phosphate or FGF23 to iron therapy. These two subjects had been iron sufficient at baseline and demonstrated, in fact, a sharp and continuous increase in serum ferritin during the study to levels over 200 μg/L, which raises concerns about iron overload if iron therapy was to be continued. The WHO defines the criteria for iron overload as serum ferritin concentrations over 150 and 200 μg/L in females and males, respectively.^14^

## Further Insights Into the Natural Course of Disease

As far as evidence goes, iron deficiency constitutes a risk to develop disease manifestations for patients with ADH and quite likely explains the time of presentation in life, the spontaneous healing, the late onset, and the relapses. Iron deficiency can occur at any time in life and hence ADH also may become symptomatic at any time. Seton & Jüppner^15^ reported a woman who was first diagnosed with ADH at age 85 years; her history indicated intermittently recurring disease from early childhood diagnosis of rickets to bone pain from osteomalacia in adulthood; although her iron status at presentation was not documented she was not anemic. Of the 11 symptomatic subjects in the Chinese series, seven were iron deficient and two further subjects were suspected to be deficient.^11^ Therefore, quite possibly, the intermittent hypophosphatemic manifestations in ADH occur in synchrony with episodes of iron deficiency. However, to date, we still know very little about modifying genes and how exactly iron deficiency stimulates FGF23 mRNA expression.

## Oral Iron Therapy: Long‐Term Observation of the Original Case

**Figure 1:**
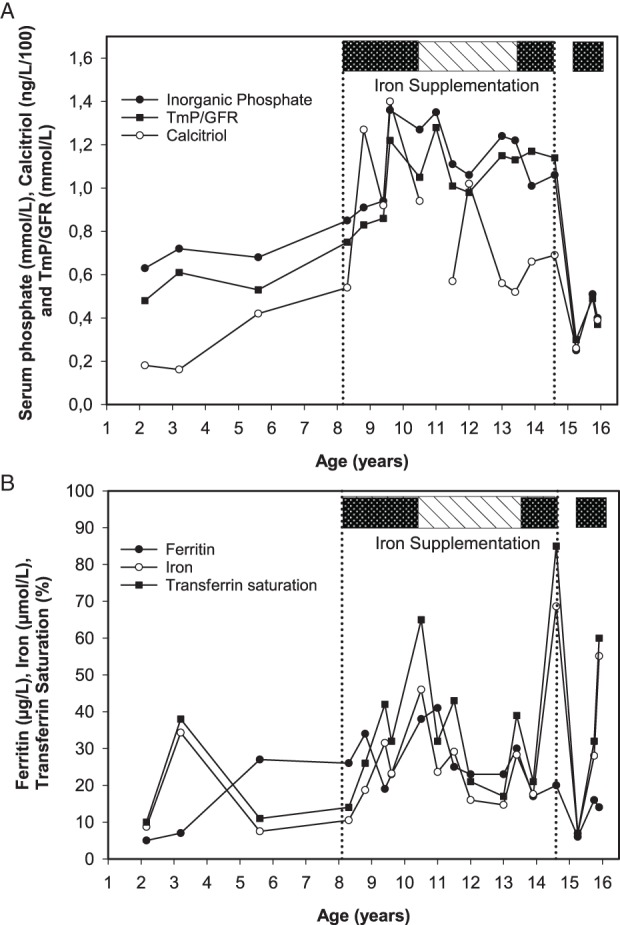
Fourteen‐year longitudinal observation of a female patient with ADH who first presented at age 26 months with rickets and iron deficiency. (*A*) Phosphorus metabolism; (*B*) the corresponding iron metabolism. Before starting oral iron, she received only conventional rickets therapy, which was fully stopped at age 9.25 years. Continuous oral iron therapy is depicted as a dark box, and 3‐month intermittent oral iron supplementation as a dashed box. The vertical lines represent start and end of iron supplementation period. Analysis of all her biochemical data points revealed that serum phosphate correlated best with ferritin (*r* = 0.65, *p* = .003) and calcitriol concentrations (*r* = 0.62, *p* = .008).

Here, we take the opportunity to share further longitudinal, observational data on the original ADH case that first demonstrated the reversibility of hypophosphatemia on oral iron therapy.^12^ The girl had presented with hypophosphatemic rickets and iron deficiency at age 26 months and was found to have a typical R179Q mutation in *FGF23*. Rickets responded well to conventional therapy. Only after high‐dose oral iron therapy (3.5 mg/kg/day) from age 8.1 years did her phosphate metabolism normalize so that all rickets medication could be withdrawn. As seen in Fig. 1 *A,B* her serum phosphorus remained normal while she was taking oral iron supplements intermittently as per WHO guidelines^16^, ^17^ during her early pubertal years, although her compliance was also erratic and her ferritin remained relatively low. From her 14th birthday, just before her menarche at age 14.2 years, she was taking regular supplemental iron (II) sulfate at a dose of 2.3 mg/kg/day (below the therapeutic dose of 3 to 6 mg/kg of elemental iron), and at her next visit 8 months later her serum iron concentration was 68.6 μmol/L (383 μg/dL), transferrin saturation 85% (7% to 46%), levels possibly associated with iron toxicity, while her ferritin was 20 μg/L.^18^ Unconfirmed suspicion arose that she may have deliberately taken higher doses prior to the consultation, presumably to hide noncompliance; her iron supplements were nevertheless stopped for safety reasons.

Eight months later, she presented with polymenorrhea, severe hypophosphatemia (serum phosphate 0.25 mmol/L), and iron deficiency without anemia (iron 6.9 μmol/L, transferrin saturation 7%, ferritin 6 μg/L). She was once again restarted on oral iron supplements (2 mg/kg/day) and serum phosphorus and renal tubular phosphate reabsorption in mass per unit volume of glomerular filtrate (TmP/GFR) improved. Nonetheless, polymenorrhea persisted and to date, age 16 years, neither serum phosphorus nor ferritin have normalized on supplementation despite improvement in iron status (Fig. 1), presumably due to insufficient iron dose in relation to heavy menstrual blood loss, or compliance.

Spearman correlation analysis of all available biochemical data over the 14‐year observation period was performed to assess the relationship of phosphate and iron metabolism. The results show that serum phosphate was most closely correlated to serum ferritin (*r* = 0.65, *p* = .003) and calcitriol (*r* = 0.62, *p* = .008), not with serum iron or transferrin saturation (*r* = not significant [n.s.]). Similarly, TmP/GFR showed significant correlation only with ferritin (*r* = 0.54, *p* = .02) and calcitriol (*r* = 0.62, *p* = .008).

Overall, evidence from these longitudinal observations clearly indicates that iron deficiency triggers hypophosphatemia in ADH, and that oral iron treatment reverses deficiencies in both elements. In normophosphatemic ADH subjects, iron had no effect on phosphate metabolism in the study by Imel and colleagues,^13^ so the direct correlation between elements *only* exists during iron deficiency, which suggests that the lack of iron must be the trigger to increased FGF23 mRNA expression. The fact that most symptomatic patients are women during reproductive age (90% in the Chinese cohort)^11^ suggests that even mild iron deficiency without anemia (ie, from hypermenorrhea or polymenorrhea) serves as a trigger for FGF23 excess in ADH.

## Oral Iron for Prevention and Treatment: the New Management Strategy for ADH

Given this new evidence, what is the way forward in the management of ADH patients? Iron deficiency is the most frequent micronutrient deficiency globally, affecting one‐third of non‐pregnant women.^17^ Certain risk groups, such as individuals with ADH, need to be regarded as at high risk of complications. The strategy will undoubtedly have to be the avoidance and treatment of iron deficiency, in order to maintain or re‐establish normophosphatemia in ADH patients. Hence, in our view there are three distinct groups of patients carrying *FGF23* mutations, judged by their ferritin status, with different proposed management criteria (see Table 1):

- The first group constitutes normophosphatemic and iron‐sufficient ADH patients who require regular monitoring of both elements but no regular iron supplements.
- The second group are normophosphatemic patients at risk of iron deficiency, specifically all women during the reproductive age, people on vegetarian or vegan diets, or with malabsorptive or cardiac conditions, where preventative supplementation doses of oral iron are indicated. These should follow the WHO guidance on iron supplementation.^16^, ^17^
- The third group consists of hypophosphatemic ADH patients who require oral iron in treatment doses (3 to 6 mg/kg of elemental iron) until normophosphatemia is reached, followed by the WHO supplementation regimen.

**Table 1:** Clinical Management of ADH Patients Depending on Iron Status

ADH risk groups	Management	Goal
Low‐risk, normophosphatemic, normal ferritin (iron‐sufficient)	Only monitoring	Maintain normal ferritin levels
At risk, normophosphatemic, iron sufficient or deficient, including women of reproductive age	Oral iron supplementation (1‐2 mg/kg of elemental Fe) for 3 months/year	Maintain/reach normal ferritin levels, avoid iron overload
Hypophosphatemic, iron deficient (with/without anemia), low ferritin	Oral iron treatment (3 to 6 mg/kg elemental Fe, max 200 mg/day) for 3 months, followed by supplementation	Reach normophosphatemia, avoid iron toxicity and overload

Oral iron therapy needs to be handled with caution. Careful and regular monitoring of iron status is therefore recommended in subjects with ADH, to avoid iron toxicity^18^ and overload.^14^ During phases of hypophosphatemia, active vitamin D analogues and oral phosphate can of course be given alongside iron therapy. For subjects who do not tolerate or accept iron substitution, both conventional therapy as well as burosumab are available treatment options. As for all forms of hypomineralization, 25OH vitamin D should be kept in the normal range.

Evidence from the medical literature on oral iron therapy in ADH,^11^, ^12^, ^13^ including our long‐term observation presented here, indicate that ferritin, a marker of the body's iron store, may be the best indicator relevant to medical decision making. We therefore propose ferritin as the main monitoring parameter by which to judge the need for iron supplementation in ADH.

## A Glimpse Into the Future

Preventing the manifestation of a genetic disease may appear to be a strange concept specific to ADH. We propose that the maintenance of normal iron status, assessed by serum ferritin, should be the main monitoring target in subjects with known ADH. If the existing evidence gained from the few kindreds worldwide is corroborated, then prevention of hypophosphatemia with associated rickets and osteomalacia should be possible.

For those newly diagnosed or with a relapse whose iron stores are low (low ferritin, iron‐deficiency anemia), the first line of therapy should be oral iron because a reversal of the phenotype and reaching normophosphatemia should be possible if oral iron therapy is tolerated and taken. Treatment with active vitamin D analogues and oral phosphate may be reserved to those not accepting or tolerating oral iron. Intravenous iron preparations, specifically ferric carboxymaltose, can cause severe and prolonged hypophosphatemia and osteomalacia^19^ and thus should be avoided in ADH.

For an ultrarare disease such as ADH, evidence and clinical experience is hard to come by. International registries for rare bone diseases are the only way forward to gain systematic new evidence from patients. We call on the international community to join these registries.

## Disclosures

All authors state that they have no conflicts of interest.
